# GM1 Oligosaccharide Modulates Microglial Activation and α-Synuclein Clearance in a Human In Vitro Model

**DOI:** 10.3390/ijms26157634

**Published:** 2025-08-07

**Authors:** Giulia Lunghi, Carola Pedroli, Maria Grazia Ciampa, Laura Mauri, Laura Rouvière, Alexandre Henriques, Noelle Callizot, Benedetta Savino, Maria Fazzari

**Affiliations:** 1 Department of Medical Biotechnology and Translational Medicine, Università degli Studi di Milano, 20054 Segrate, Italy; giulia.lunghi@unimi.it (G.L.); carola.pedroli@studenti.unimi.it (C.P.); maria.ciampa@unimi.it (M.G.C.); laura.mauri@unimi.it (L.M.); benedetta.savino@unimi.it (B.S.); 2 Neuro-Sys, 410 Chemin Départemental 60, 13120 Gardanne, France; laura.rouviere@neuro-sys.com (L.R.); alexandre.henriques@neuro-sys.com (A.H.); noelle.callizot@neuro-sys.com (N.C.)

**Keywords:** GM1 oligosaccharide, microglia, neurodegeneration, Parkinson’s disease, α-synuclein

## Abstract

Neuroinflammation driven by microglial activation and α-synuclein (αSyn) aggregation is one of the central features driving Parkinson’s disease (PD) pathogenesis. GM1 ganglioside’s oligosaccharide moiety (OligoGM1) has shown neuroprotective potential in PD neuronal models, but its direct effects on inflammation remain poorly defined. This study investigated the ability of OligoGM1 to modulate microglial activation and αSyn handling in a human in vitro model. Human embryonic microglial (HMC3) cells were exposed to αSyn pre-formed fibrils (PFFs) in the presence or absence of OligoGM1. Microglial activation markers, intracellular αSyn accumulation, and cytokine release were assessed by immunofluorescence and ELISA. OligoGM1 had no effect on microglial morphology or cytokine release under basal conditions. Upon αSyn challenge, cells exhibited increased amounts of ionized calcium-binding adaptor molecule 1 (Iba1), triggered receptor expressed on myeloid cells 2 (TREM2), elevated αSyn accumulation, and secreted pro-inflammatory cytokines. OligoGM1 pre-treatment significantly reduced the number and area of Iba1(+) cells, the intracellular αSyn burden in TREM2(+) microglia, and the release of interleukin 6 (IL-6). OligoGM1 selectively attenuated αSyn-induced microglial activation and enhanced αSyn clearance without compromising basal immune function. These findings confirm and support the potential of OligoGM1 as a multitarget therapeutic candidate for PD that is capable of modulating glial reactivity and neuroinflammatory responses.

## 1. Introduction

Parkinson’s disease (PD) is the second most common neurodegenerative disorder worldwide, characterized by the progressive degeneration of dopaminergic neurons in the substantia nigra pars compacta combined with the pathological accumulation of misfolded α-synuclein (αSyn) aggregates [1,2,3]. Dopaminergic neuronal loss is accompanied by mitochondrial dysfunction, oxidative stress, lysosomal impairment, neurochemical imbalance, and reduced neurotrophic support [4]. Additionally, accumulating evidence implicates chronic neuroinflammation as a key driver in PD pathogenesis [5,6].

Indeed, αSyn oligomers and pre-formed fibrils (PFFs) promote the sustained activation microglia, the resident immune cells of the central nervous system, that contribute to disease progression by releasing pro-inflammatory cytokines and reactive oxygen species, thereby exacerbating neuronal degeneration [2,4,7,8,9,10]. Thus, therapeutic strategies aimed at reducing αSyn aggregation while dampening microglial activation are therefore of considerable interest in the quest for disease-modifying interventions for PD.

Ganglioside GM1, a major component of neuronal membranes, has long been recognized for its neuroprotective, antioxidant, anti-apoptotic, and anti-inflammatory properties [2,4,11]. However, its therapeutic potential is limited by its amphiphilic nature, which severely restricts its ability to cross the blood–brain barrier. Recent research has identified the oligosaccharide portion of GM1 (OligoGM1) as its pharmacologically active moiety [12]. OligoGM1 retains the neuroprotective properties of GM1 by engaging Trk receptors at the neuronal surface while displaying favorable pharmacokinetic characteristics, including efficient blood–brain barrier penetration due to its small, hydrophilic structure. Beyond its established neuroprotective effects, our recent work demonstrated that OligoGM1 reduces αSyn accumulation and protects against mitochondrial dysfunction and lysosomal overload in a cell model of genetic PD [13,14,15,16].

Preclinical studies suggest that OligoGM1 may also exert anti-inflammatory effects in glial cells. In both in vitro and in vivo PD models, OligoGM1 reduced the αSyn-induced activation of microglia cultured with neurons, suggesting its potential to modulate neuroinflammation [14,15]. However, a characterization of its direct impact on microglia, the fundamental immune cells in the central nervous system, particularly under αSyn-induced inflammatory stress, remains to be elucidated.

The present study aimed to determine whether OligoGM1 can attenuate microglial activation and promote a homeostatic, potentially neuroprotective phenotype, thus extending its therapeutic relevance to the regulation of neuroinflammation in PD. To this end, we investigated the ability of OligoGM1 to modulate αSyn-induced inflammatory responses in immortalized human embryonic microglial cells (HMC3) [17]. Specifically, we assessed microglial activation markers [ionized calcium-binding adaptor molecule 1 (Iba1), triggering receptor expressed on myeloid cells 2 (TREM2)], intracellular αSyn accumulation, and the release of pro-inflammatory cytokines following exposure to αSyn PFFs.

Our results demonstrate that OligoGM1 does not alter the physiological state of microglial cells, supporting its favorable safety and translational profile. Conversely, in αSyn-challenged HMC3 cells characterized by a pro-inflammatory phenotype with increased Iba1(+) and TREM2(+) cell number and area and an elevated secretion of tumor necrosis factor alpha (TNF-α) and interleukin 6 (IL-6), OligoGM1 effectively attenuated αSyn-induced alterations by reducing Iba1(+) cell area and limiting αSyn accumulation within TREM2(+) cells without broadly altering cytokine release. These findings highlight the ability of OligoGM1 to modulate microglial reactivity and support its potential as a therapeutic candidate for the treatment of neuroinflammation in PD.

## 2. Results

### 2.1. Maintenance of Microglial Resting Conditions upon OligoGM1 Treatment

HMC3 microglial cells represent a well-characterized model of human microglia and are thus commonly used to study neuroinflammation [17,18]. These cells express canonical microglial markers such as Iba1 and TREM2, and they can produce a broad range of pro-inflammatory cytokines, including TNF-α and IL-6. Based on these features, we selected this model to investigate the effect of OligoGM1 in an inflammatory context.

Previous studies in both in vitro and in vivo systems have demonstrated that OligoGM1 has an anti-inflammatory effect in mixed neuron-glia cultures, suggesting its potential to modulate neuroinflammatory responses [14].

As a first step, we evaluated the impact of OligoGM1 on microglial activation and inflammatory response under basal (non-injured) conditions. HMC3 cells were exposed to OligoGM1 (100 µM), and the endpoints analyzed included Iba1 and TREM2 immunoreactivity, as well as the levels of secreted TNF-α and IL-6.

**Figure 1:**
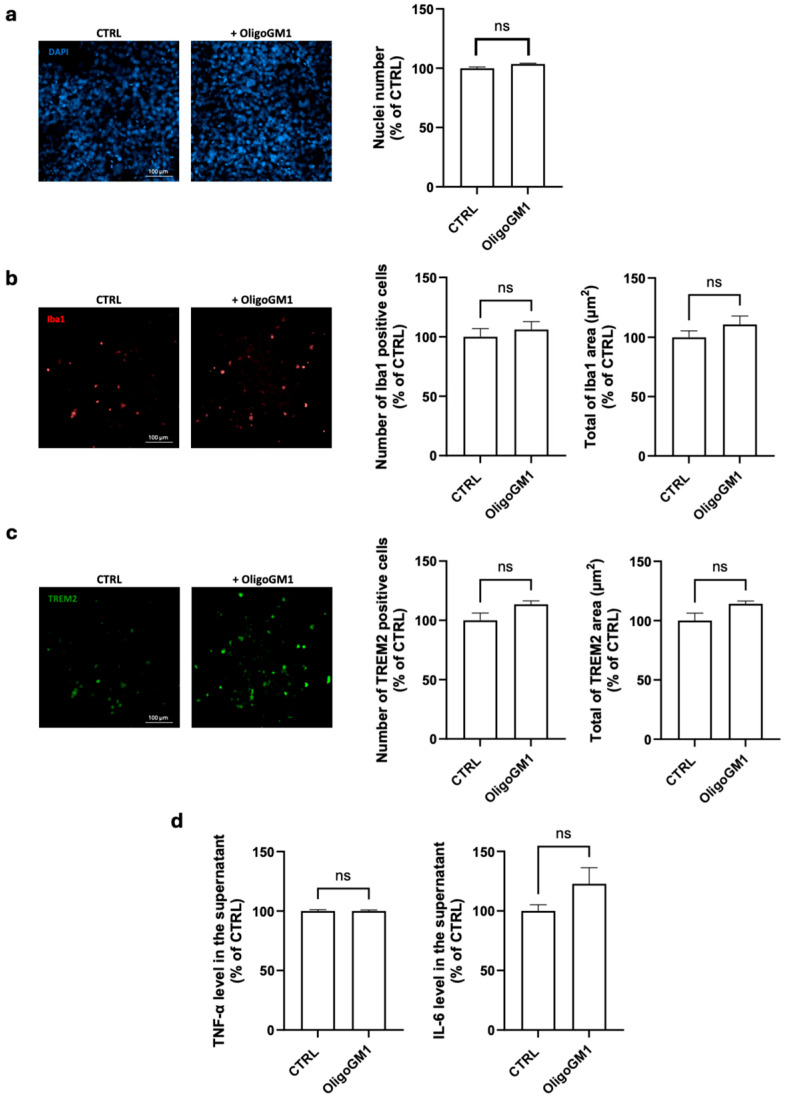
Effects of GM1 oligosaccharide (OligoGM1) application on Human embryonic microglial (HCM3) cells. After 2 days of culture (80% of confluence), HCM3 cells were incubated or not incubated (control, CTRL) with OligoGM1 (100 µM). After 48 h, immunofluorescence staining [(against nuclei, ionized calcium-binding adaptor molecule 1 (Iba1) and triggered receptor expressed on myeloid cells 2 (TREM2)] and ELISA for quantification of released cytokines to medium were performed, as described in the Section 4: (**a**) Representative immunofluorescence images (20× magnification, scale bar 100 µm) and number of DAPI-positive cells; (**b**) Representative immunofluorescence images (20× magnification, scale bar 100 µm) of Iba1 expression, quantification of Iba1 accumulation (Iba1 area normalized by the number of cells), and area of Iba1-positive cells (area of microglia cells normalized by the µm^2^ of Iba1); (**c**) Representative immunofluorescence images (20× magnification, scale bar 100 µm) of TREM2 expression, quantification of TREM2-positive cells (TREM2 area normalized by the number of cells), and area of TREM2-positive cells (area of microglia cells normalized by the µm^2^ of TREM2); (**d**) Quantification of released cytokines tumor necrosis factor alpha (TNF-α) and interleukin 6 (IL-6). All values are represented as percentage versus CTRL and expressed as mean ± standard error of the mean (SEM, *n* = 6; ns = not significant, Mann–Whitney test).

Under basal conditions, OligoGM1 treatment did not significantly affect cell viability or the number or area of Iba1(+) or TREM2(+) cells (Figure 1a–c), indicating that OligoGM1 does not exert cytotoxic effects, nor does it induce microglial activation. Additionally, no significant changes were observed in TNF-α or IL-6 release (Figure 1d). Importantly, these data suggest that OligoGM1 in a physiological context does not alter or stimulate the inflammatory microglia environment, further supporting its translational potential for clinical application in the treatment of complex neurodegenerative diseases.

### 2.2. Protective Effects of OligoGM1 on αSyn-Induced Microglia Activation

Having established that OligoGM1 did not elicit any activation of microglia under basal conditions and lacked intrinsic immunogenic properties, we next explored its potential protective effects under a pro-inflammatory challenge, specifically by using αSyn as an inflammatory stimulus. αSyn oligomers and PFFs are known to exert cytotoxic effects through various intracellular mechanisms, including mitochondrial and endoplasmic reticulum stress, and the impairment of the autophagy–lysosomal system [19,20,21,22,23,24]. In addition, αSyn can propagate between neurons and glial cells in a prior-like, self-amplifying manner, contributing to disease progression across brain regions [19].

We have previously demonstrated that OligoGM1 reduces αSyn-induced microglial activation in mixed neuron-glia cultures [14] and attenuates lysosomal and mitochondrial dysfunctions in SH-SY5Y glucocerebrosidase-mutant [13]. However, its direct effects on microglia have not been specifically evaluated in an isolated model.

**Figure 2:**
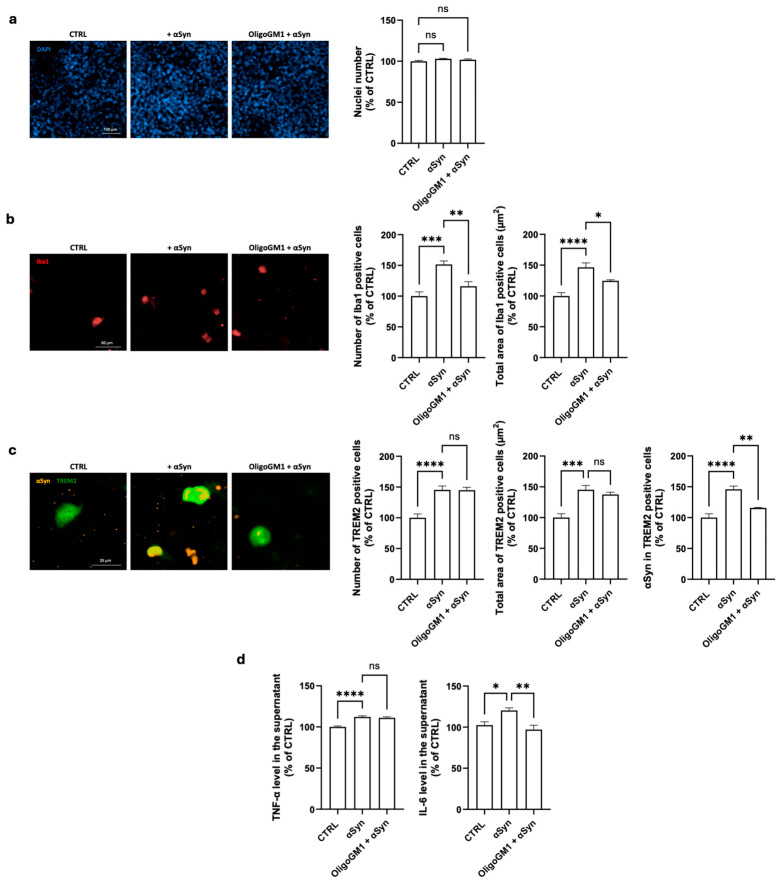
OligoGM1 modulation of αSyn-induced activation of HMC3 cells. After 2 days of culture (80% of confluence), HCM3 cells were incubated or not incubated (CTRL) with OligoGM1 (100 µM). After 4 h, αSyn (1 µM) was added to the culture medium for 48 h. At the end of treatment, immunofluorescence staining (against nuclei, Iba1, TREM2, and αSyn) and IL-6/TNF-α quantification from medium were performed as described in the Section 4. (**a**) Representative immunofluorescence images (20× magnification, scale bar 100 µm) and number of DAPI-positive cells; (**b**) Representative immunofluorescence images (20× magnification, scale bar 50 µm) of Iba1 expression, quantification of Iba1 accumulation (Iba1 area normalized by the number of cells), and area of Iba1-positive cells (area of microglia cells normalized by the µm^2^ of Iba1); (**c**) Representative immunofluorescence images (20× magnification, scale bar 25 µm) of TREM2 and αSyn expressions, quantification of TREM2-positive cells (TREM2 area normalized by the number of cells), area of TREM2-positive cells (area of microglia cells normalized by the µm^2^ of TREM2), and quantification of αSyn area accumulated within TREM(+) cells (area of αSyn normalized by the µm^2^ of TREM2); (**d**) Quantification of released cytokines TNF-α and IL-6. All values are represented as percentage versus CTRL and expressed as mean ± SEM (*n* = 6; * *p* < 0.05, ** *p* < 0.01, *** *p* < 0.001, **** *p* < 0.0001, ns = not significant by one-way ANOVA followed by Fisher’s LSD). * *p* < 0.05 was considered significant.

To this purpose, HMC3 microglial cells were exposed to αSyn alone (1 µM, 48 h). This treatment did not affect cell viability, as the total cell number remained unchanged (Figure 2a). However, this treatment led to a marked increase in both the number and the area of Iba1(+) and TREM2(+) cells (Figure 2b,c) that was consistent with significant microglial activation. Moreover, αSyn accumulated within TREM2(+) cells (Figure 2c), indicating enhanced phagocytic uptake. In parallel, αSyn treatment significantly elevated the release of pro-inflammatory cytokines TNF-α and IL-6 (Figure 2d), confirming the activation of an inflammatory response.

Pre-treatment with OligoGM1 (4 h prior to αSyn exposure) significantly counteracted several αSyn-induced alterations. Specifically, OligoGM1 significantly reduced both the number and area of Iba1(+) cells, indicating a partial inhibition of microglial activation (Figure 2b). Importantly, the number and area of TREM2(+) cells remained unchanged following OligoGM1 treatment (Figure 2c), supporting the maintenance of the alternatively activated M2 phenotype of microglial cells [25,26]. OligoGM1 also significantly decreased the accumulation of intracellular αSyn within TREM2(+) cells (Figure 2c), restoring levels closer to baseline. While TNF-α levels remained unaffected, OligoGM1 pre-treatment lowered IL-6 release in αSyn-injured cells (Figure 2d).

These findings suggest that OligoGM1 confers a protective effect on microglial cells challenged with αSyn by reducing their activation state and promoting αSyn clearance while selectively modulating the release of inflammatory mediators.

## 3. Discussion

Chronic neuroinflammation is now recognized as a central pathological feature of PD, driven in large part by microglial activation in response to misfolded αSyn aggregates. The present study provides new insights into the modulatory role of OligoGM1—the hydrophilic, brain-permeable portion of GM1 ganglioside—on microglial responses under neuroinflammatory conditions relevant to PD, expanding its previously known neuroprotective and anti-aggregative properties [14,15,27,28].

Our data show that OligoGM1 did not induce microglial activation and preserved a homeostatic state, as it did not modify the expression of canonical microglial markers (Iba1, TREM2) or increase the release of pro-inflammatory cytokines (TNF-α, IL-6), supporting its non-immunogenic profile (Figure 1). However, when microglial HMC3 cells were exposed to αSyn PFFs, they exhibited increased Iba1 expression, morphological changes, and cytokine release, typical hallmarks of activation (Figure 2). OligoGM1 pre-treatment mitigated these effects, particularly reducing αSyn accumulation and cell activation without fully suppressing cytokine production (Figure 2). This suggests a selective immunomodulatory action that preserves microglia surveillance functions, a quality increasingly emphasized in neuroinflammation research as essential for maintaining the homeostasis of the central nervous system [7,29]. These findings are consistent with earlier observations in a co-culture system of neurons and microglia where OligoGM1 mitigated αSyn-driven microglial activation and neuronal injury [14]. Importantly, OligoGM1 has been shown to directly prevent αSyn aggregation through the structural stabilization of monomeric conformers, thereby limiting the availability of toxic oligomers and hindering the seeding process. The current study supports a complementary mechanism, showing that OligoGM1 facilitated intracellular αSyn clearance, particularly in TREM2(+) microglia, which are known to coordinate phagocytic and metabolic responses to neuronal damage [30]. It has been demonstrated that TREM2 deficiency worsens αSyn pathology and exacerbates neuroinflammation by reprogramming the microglia from a protective to a proinflammatory state [24], and, in accordance, TREM2 mutations can increase susceptibility to sporadic PD [31]. Recent data also point to OligoGM1’s ability to restore lysosomal-autophagic flux in a GBA-mutant neuronal model [13]. This is especially relevant considering the observed decrease in αSyn levels in TREM2(+) cells upon OligoGM1 treatment, suggesting enhanced degradative capability. Since TREM2 is emerging as a master regulator of microglial phenotype, modulating PI3K-AKT-mTOR pathways and phagocytosis [19,32], future studies should confirm whether OligoGM1 targets the downstream effectors of TREM2 to shift microglial polarization toward a reparative state. Indeed, parallel evidence from in vivo and neuronal models has highlighted the ability of OligoGM1 to enhance neuronal survival, restore mitochondrial function, and limit oxidative damage in MPTP-intoxicated neurons via the activation of AKT/mTOR signaling [15]. Given the critical interplay between mitochondrial dysfunction, αSyn pathology, and neuroinflammation [19,32], our present findings extend OligoGM1’s therapeutic relevance beyond neurons to microglial cells, which are equally affected by metabolic stress and impaired mitophagy in PD.

While HCM3 cells maintain key antigenic signatures and express several microglia-specific markers, we acknowledge that OligoGM1’s immunomodulatory and degradative potential may be underestimated due to the intrinsic limitations of this model. In particular, HMC3 cells show attenuated and narrower cytokine responses, especially in resting conditions, with some pro- and anti-inflammatory molecules (e.g., IFNγ, IL-1α, IL-1β, IL-4, IL-10, TGF-β1/2) being absent or only marginally detectable [17,33,34,35,36,37].

Furthermore, their phagocytic activity is lower than that of primary microglia [17,18], potentially limiting the assessment of OligoGM1’s impact on clearance pathways [13]. This is supported by evidence from Akhter et al. showing that TREM2, a key phagocytic marker, is expressed at lower levels in HMC3 cells, though it is upregulated upon amyloid-β42 exposure [38]. Accordingly, we observed a low basal percentage (1.77% ± 0.13%) of TREM2(+) cells that increased upon αSyn stimulation (2.47 ± 0.1%). OligoGM1 treatment did not reduce the number of TREM2(+) cells over total cells (2.5 ± 0.08%) when compared to αSyn-treated samples, but it significantly enhanced their capability of degrading internalized αSyn, suggesting an improvement in functional phagocytic activity. This finding is particularly relevant given TREM2’s key role in shifting microglia from a pro-inflammatory M1 phenotype toward a reparative, neuroprotective M2 state [25,26].

On the other hand, due to their human origin and reproducibility, HMC3 cells provide a translationally relevant in vitro platform that reliably models key aspects of αSyn-induced microglial activation, including Iba1 and TREM2 upregulation, intracellular αSyn accumulation, and the induction of IL-6 and TNF-α secretion. The relevance of using human microglial models such as HMC3 is underscored by increasing evidence that human and rodent microglia differ significantly in inflammatory responses and metabolic programming [39]. Additionally, our data are corroborated by previous reports using mixed primary cultures, enabling microglia–neurons crosstalk and highlighting translational consistency [14].

In summary, the present work supports the multifaceted potential of OligoGM1 in the treatment of multifactorial PD. Its dual activity in blocking αSyn aggregation and modulating microglial reactivity without basal immune suppression positions it as a promising disease-modifying agent. The restoration of a homeostatic, non-cytotoxic, microglial phenotype could significantly impact PD progression, especially in early phases where neuroinflammation is a major driver. However, validation in human iPSC-derived systems and animal models of synucleinopathy will be necessary to confirm the translational potential of OligoGM1 in modulating glial responses.

Collectively, our findings contribute to a growing body of literature supporting OligoGM1 as a pleiotropic molecule capable of intervening at multiple levels of PD pathogenesis, including αSyn proteostasis, mitochondrial resilience, autophagy, and neuroimmune balance.

## 4. Materials and Methods

### 4.1. Materials

Commercial chemicals were of the highest purity available, common solvents were distilled before use, and water was doubly distilled in a glass apparatus.

Phosphate-buffered saline (PBS) and Calcium Magnesium Free-PBS, glucose, RNAase-free water, paraformaldehyde (PFA), sodium orthovanadate (Na_3_VO_4_), bovine serum albumin (BSA), ethylenediaminetetraacetic acid (EDTA), Trypsin, sodium dodecyl sulfate (SDS), high-performance thin layer chromatography (HPTLC), and DPX Mountant (06522) were obtained from Sigma-Aldrich (St. Louis, MO, USA).

Fetal Bovine Serum (FBS), L-Glutamine, and penicillin/streptomycin (P/S) solution were obtained from EuroClone (Paignton, UK).

The human αSyn (R-peptide) for cell culture was obtained from Watkinsville (Watkinsville, GA, USA).

The Hoechst solution (33342), Human TNF-α (KHC3011), and the IL-6 (EH21L6) ELISA Kit were obtained from Thermo Fischer Scientific (Waltham, MA, USA).

Eagle’s minimum essential medium (EMEM) was obtained from Life Technologies (St. Christophe, France).

### 4.2. Antibodies

For immunofluorescence analyses, the following antibodies were used: primary mouse monoclonal anti-TREM2 antibody [Cat. 11084-MM08, research resource identifier (RRID):AB_2860315], purchased from Sino Biological Europe GmbH, Europe (Düsseldorfer, Germany); primary rabbit polyclonal anti-αSyn antibody (Cat. 2642S, RRID not available), purchased from Ozyme, Paris, France; and primary goat polyclonal anti-Iba1 antibody (Cat. Ab5076, RRID:AB_2224402), purchased from Abcam, Cambridge, UK.

These antibodies were revealed with secondary antibodies: donkey anti-rabbit IgG coupled with an Alexa Fluor 568 (CF568); donkey anti-goat IgG coupled with an Alexa Fluor 488 (CF488); and donkey anti-goat IgG coupled with an Alexa Fluor 647, all purchased from Sigma Aldrich (St. Luis, MO, USA),

### 4.3. Culture of HMC3 Cell Line

Cryopreserved HMC3 cells obtained from ATCC (CRL-3304) were rapidly thawed in a water bath at 37 °C. Cells were transferred to a tube containing a defined culture medium (complete growth medium) consisting of EMEM and 10% FBS and centrifuged 515× *g* for 5 min at 23 °C. Cell pellets were suspended in EMEM supplemented with 10% FBS, 1% P/S solution, and 1% glutamine.

Cells were seeded in a 96-well plate at a density of 15,000 cells per well and were cultured at 37 °C in an air (95%)-CO_2_ (5%) incubator.

### 4.4. Cell Treatments

After 2 days of culture, when cells reached 80% of confluence, OligoGM1 was dissolved in culture medium and administered to HMC3 cells at a final concentration of 100 µM. A concentration of 100 μM OligoGM1 was selected based on previous studies in primary neuronal cultures—including granule and dopaminergic—as well as in mixed dopaminergic neuron–microglia co-cultures, where it consistently exhibited neuritogenic, neuroprotective, and anti-inflammatory properties [14,15]. Where indicated, 4 h after OligoGM1 incubation, αSyn was added at a final concentration of 1 µM. CTRL cells were incubated under the same experimental conditions but received a vehicle (H_2_O) instead of OligoGM1 and α-syn.

### 4.5. Cell Immunostaining

After 48 h of OligoGM1 treatment, in the presence or absence of α-syn, cell culture medium was removed, and the cells were washed with PBS and fixed with a solution of 4% PFA in PBS with pH = 7.3 for 20 min at 23 °C. Afterwards, cells were washed twice in PBS and then permeabilized with 0.1% saponin and 1% FBS in PBS for 15 min at 23 °C. Non-specific sites were blocked with a solution of PBS containing 5% of FBS for 15 min at 23 °C. Then, cells were incubated for 2 h at 23 °C with the following primary antibodies: (i) anti-Iba1 antibody at a dilution of 1:500 in PBS containing 1% FBS, 0.1% saponin; (ii) anti-αSyn antibody at a dilution of 1:200 in PBS containing 1% FBS, 0.1% saponin; (iii) anti-TREM2 antibody at a dilution of 1:50 in PBS containing 1% FBS, 0.1% saponin.

Primary antibodies were revealed with Alexa Fluor 488 CF488 donkey anti-mouse IgG at a dilution of 1:400 and with Alexa Fluor 568 CF568 donkey anti-rabbit IgG at a dilution of 1:400 in PBS containing 1% FBS and 0.1% saponin for 1 h at 23 °C. Nuclei were counter-stained with a DAPI dye solution (1:1000 in PBS).

For each condition, 25 pictures per well (representing the whole well area) were automatically taken using the Operetta^®^ system (Revvity, Waltham, MA, USA) at 20× magnification using the same acquisition parameters. From the images, analyses were directly and automatically performed by Harmony^®^ (Revvity).

The following read-outs were investigated: (i) the number of Iba1-positive cells; (ii) the area of Iba1-positive cells (area of microglial cells, µm^2^ of Iba1); (iii) the number of TREM2-positive cells; (iv) the area of TREM2-positive cells (area of microglial cells, µm^2^ of TREM2); (v) α-syn accumulation in M2 microglial cells in µm^2^ (overlapping between TREM2 and α-syn).

### 4.6. Quantification of TNF-α and IL-6

After 48 h of OligoGM1 treatment, in the presence or absence of α-syn, the cell culture supernatant was collected for cytokine quantification. This analysis was performed on fresh samples. The levels of pro-inflammatory cytokines were determined in the cell culture supernatant by ELISA according to the manufacturer’s instructions (Thermo Fischer Scientific, Waltham, MA, USA).

### 4.7. OligoGM1 Preparation

OligoGM1 was prepared by ozonolysis followed by the alkaline degradation of GM1 [40]. Minor changes for the alkaline degradation were introduced. Briefly, GM1 ganglioside was dissolved in the minimum amount of methanol required, and it was slowly saturated with and maintained under ozone at 23 °C for 6 h under continuous stirring. Following the solvent’s evaporation under vacuum, triethylamine was added to bring the residue’s pH between 10.5 and 11.0. Following solvent evaporation, OligoGM1 was purified by flash chromatography with the following eluent ratio: chloroform/methanol/2-propanol/water, 60:35:5:5 by vol. OligoGM1 was dissolved in sterile ultrapure water and stored at −20 °C. Nuclear magnetic resonance, mass spectrometry, and HPTLC analyses showed a purity of over 99% for the prepared oligosaccharide [28].

### 4.8. αSyn Preparation

Human αSyn peptide was reconstituted in a defined culture medium at 4 µM (stock solution) and slowly shacked at 37 °C for 3 days in darkness to generate the αSyn oligomers and PFFs (for details, see [19]).

### 4.9. Statistical Analysis

Data are expressed as mean ± standard error of the mean (SEM). Statistical analysis was performed by Mann–Whitney tests or one-way ANOVA followed by Fisher’s LSD tests using Prism software 10.3.0 (GraphPad Software, Inc., La Jolla, CA, USA; https://www.graphpad.com/ accessed on 1 July 2024)). *p* < 0.05 was considered significant.

## Data Availability

The data presented in this study are available on reasonable request to the corresponding authors.

## Abbreviations

The following abbreviations are used in this manuscript:

Ganglioside nomenclature is in accordance with IUPAC-IUBB recommendations [41]	
BSA	bovine serum albumin
CTRL	control
EDTA	ethylenediaminetetraacetic acid
EMEM	Eagle’s minimum essential medium
FBS	fetal bovine serum
GM1	II^3^Neu5Ac-GG_4_Cer, β-Gal-(1-3)-β-GalNac-(1-4)-[α-Neu5Ac-(2-3)]-β-Gal-(1-4)-β-Glc-Cer
HMC3	human embryonic microglial cells
HPTLC	high-performance thin layer chromatography
Iba1	ionized calcium-binding adaptor molecule 1
IL-6	interleukin 6
NaCl	sodium chloride
ns	not significative
OligoGM1	GM1-oligosaccharide, II^3^Neu5Ac-GG_4_, β-Gal-(1-3)-β-GalNac-(1-4)-[α-Neu5Ac-(2-3)]-β-Gal-(1-4)-Glc
PBS	phosphate-buffered saline
PCR	polymerase chain reaction
PD	Parkinson’s disease
PFA	paraformaldehyde
PFFs	pre-formed fibrils
RRID	research resource identifier
SDS	sodium dodecyl sulfate
SEM	standard error of the mean
αSyn	α-synuclein
TNF-α	tumor necrosis factor alpha
TREM2	triggering receptor expressed on myeloid cells 2

## References

[1] Dorsey E.R., Constantinescu R., Thompson J.P., Biglan K.M., Holloway R.G., Kieburtz K., Marshall F.J., Ravina B.M., Schifitto G., Siderowf A. (2007). Projected number of people with Parkinson disease in the most populous nations, 2005 through 2030. Neurology.

[2] Schneider J.S. (2023). GM1 Ganglioside as a Disease-Modifying Therapeutic for Parkinson’s Disease: A Multi-Functional Glycosphingolipid That Targets Multiple Parkinson’s Disease-Relevant Pathogenic Mechanisms. Int. J. Mol. Sci..

[3] Dorsey E.R., Sherer T., Okun M.S., Bloem B.R., Brundin P., Langston J.W. (2018). The Emerging Evidence of the Parkinson Pandemic. J. Park. Dis..

[4] Schneider J.S., Singh G., Williams C.K., Singh V. (2022). GM1 ganglioside modifies microglial and neuroinflammatory responses to alpha-synuclein in the rat AAV-A53T alpha-synuclein model of Parkinson’s disease. Mol. Cell. Neurosci..

[5] Tansey M.G., Goldberg M.S. (2010). Neuroinflammation in Parkinson’s disease: Its role in neuronal death and implications for therapeutic intervention. Neurobiol. Dis..

[6] Badanjak K., Fixemer S., Smajić S., Skupin A., Grünewald A. (2021). The Contribution of Microglia to Neuroinflammation in Parkinson’s Disease. Int. J. Mol. Sci..

[7] Guzman-Martinez L., Maccioni R.B., Andrade V., Navarrete L.P., Pastor M.G., Ramos-Escobar N. (2019). Neuroinflammation as a Common Feature of Neurodegenerative Disorders. Front. Pharmacol..

[8] Putnam G.L., Maitta R.W. (2025). Alpha synuclein and inflammaging. Heliyon.

[9] Zhang X., Yu H., Feng J. (2024). Emerging role of microglia in inter-cellular transmission of alpha-synuclein in Parkinson’s disease. Front. Aging Neurosci..

[10] Du X.Y., Xie X.X., Liu R.T. (2020). The Role of alpha-Synuclein Oligomers in Parkinson’s Disease. Int. J. Mol. Sci..

[11] Guo Z. (2023). Ganglioside GM1 and the Central Nervous System. Int. J. Mol. Sci..

[12] Chiricozzi E., Di Biase E., Lunghi G., Fazzari M., Loberto N., Aureli M., Mauri L., Sonnino S. (2021). Turning the spotlight on the oligosaccharide chain of GM1 ganglioside. Glycoconj. J..

[13] Lunghi G., Pedroli C., Tagliabue I., Dobi D., Ciampa M.G., Mauri L., Rouvière L., Henriques A., Callizot N., Sonnino S. (2025). GM1 oligosaccharide-mediated rescue in GBA-linked Parkinson’s disease via modulation of lysosomal and mitochondrial dysfunctions. Glycoconj. J..

[14] Fazzari M., Di Biase E., Zaccagnini L., Henriques A., Callizot N., Ciampa M.G., Mauri L., Carsana E.V., Loberto N., Aureli M. (2023). GM1 oligosaccharide efficacy against alpha-synuclein aggregation and toxicity in vitro. Biochim Biophys. Acta Mol. Cell Biol. Lipids.

[15] Fazzari M., Lunghi G., Henriques A., Callizot N., Ciampa M.G., Mauri L., Prioni S., Carsana E.V., Loberto N., Aureli M. (2023). GM1 Oligosaccharide Efficacy in Parkinson’s Disease: Protection against MPTP. Biomedicines.

[16] Abou-Sleiman P.M., Muqit M.M., Wood N.W. (2006). Expanding insights of mitochondrial dysfunction in Parkinson’s disease. Nat. Rev. Neurosci..

[17] Dello Russo C., Cappoli N., Coletta I., Mezzogori D., Paciello F., Pozzoli G., Navarra P., Battaglia A. (2018). The human microglial HMC3 cell line: Where do we stand? A systematic literature review. J. Neuroinflamm..

[18] Janabi N., Peudenier S., Héron B., Ng K.H., Tardieu M. (1995). Establishment of human microglial cell lines after transfection of primary cultures of embryonic microglial cells with the SV40 large T antigen. Neurosci. Lett..

[19] Henriques A., Rouvière L., Giorla E., Farrugia C., El Waly B., Poindron P., Callizot N. (2022). Alpha-Synuclein: The Spark That Flames Dopaminergic Neurons, In Vitro and In Vivo Evidence. Int. J. Mol. Sci..

[20] Ballabio A., Gieselmann V. (2009). Lysosomal disorders: From storage to cellular damage. Biochim. Biophys. Acta.

[21] Nixon R.A. (2013). The role of autophagy in neurodegenerative disease. Nat. Med..

[22] Nixon R.A., Yang D.S., Lee J.H. (2008). Neurodegenerative lysosomal disorders: A continuum from development to late age. Autophagy.

[23] Zhang W., Wang T., Pei Z., Miller D.S., Wu X., Block M.L., Wilson B., Zhang W., Zhou Y., Hong J.-S. (2005). Aggregated alpha-synuclein activates microglia: A process leading to disease progression in Parkinson’s disease. FASEB J..

[24] Guo Y., Wei X., Yan H., Qin Y., Yan S., Liu J., Zhao Y., Jiang F., Lou H. (2019). TREM2 deficiency aggravates alpha-synuclein-induced neurodegeneration and neuroinflammation in Parkinson’s disease models. FASEB J..

[25] Cui W., Sun C., Ma Y., Wang S., Wang X., Zhang Y. (2020). Inhibition of TLR4 Induces M2 Microglial Polarization and Provides Neuroprotection via the NLRP3 Inflammasome in Alzheimer’s Disease. Front. Neurosci..

[26] Zhang Y., Zhang Y., Feng S., Nie K., Li Y., Gao Y., Gan R., Wang L., Li B., Sun X. (2018). TREM2 modulates microglia phenotypes in the neuroinflammation of Parkinson’s disease. Biochem. Biophys. Res. Commun..

[27] Chiricozzi E., Maggioni M., DI Biase E., Lunghi G., Fazzari M., Loberto N., Elisa M., Scalvini Grassi F., Tedeschi G., Sonnino S. (2019). The Neuroprotective Role of the GM1 Oligosaccharide, II(3)Neu5Ac-Gg4, in Neuroblastoma Cells. Mol. Neurobiol..

[28] Chiricozzi E., Chiricozzi E., Mauri L., Lunghi G., Di Biase E., Fazzari M., Maggioni M., Valsecchi M., Prioni S., Loberto N. (2019). Parkinson’s disease recovery by GM1 oligosaccharide treatment in the B4galnt1(+/−) mouse model. Sci. Rep..

[29] Koronyo-Hamaoui M., Gaire B.P., Frautschy S.A., Alvarez J.I. (2022). Editorial: Role of Inflammation in Neurodegenerative Diseases. Front. Immunol..

[30] Shi Q., Gutierrez R.A., Bhat M.A. (2025). Microglia, Trem2, and Neurodegeneration. Neuroscientist.

[31] Rayaprolu S., Rayaprolu S., Mullen B., Baker M., Lynch T., Finger E., Seeley W.W., Hatanpaa K.J., Lomen-Hoerth C., Kertesz A. (2013). TREM2 in neurodegeneration: Evidence for association of the p.R47H variant with frontotemporal dementia and Parkinson’s disease. Mol. Neurodegener..

[32] Lin D., Zhang H., Zhang J., Huang K., Chen Y., Jing X., Tao E. (2023). alpha-Synuclein Induces Neuroinflammation Injury through the *IL6ST-AS*/STAT3/HIF-1alpha Axis. Int. J. Mol. Sci..

[33] Lindberg C., Hjorth E., Post C., Winblad B., Schultzberg M. (2005). Cytokine production by a human microglial cell line: Effects of beta-amyloid and alpha-melanocyte-stimulating hormone. Neurotox. Res..

[34] Ambrosius B., Faissner S., Guse K., von Lehe M., Grunwald T., Gold R., Grewe B., Chan A. (2017). Teriflunomide and monomethylfumarate target HIV-induced neuroinflammation and neurotoxicity. J. Neuroinflamm..

[35] Rajalakshmy A.R., Malathi J., Madhavan H.N., Srinivasan B., Iyer G.K. (2014). Hepatitis C virus core and NS3 antigens induced conjunctival inflammation via toll-like receptor-mediated signaling. Mol. Vis..

[36] Hjorth E., Zhu M., Toro V.C., Vedin I., Palmblad J., Cederholm T., Freund-Levi Y., Faxen-Irving G., Wahlund L.-O., Basun H. (2013). Omega-3 fatty acids enhance phagocytosis of Alzheimer’s disease-related amyloid-beta42 by human microglia and decrease inflammatory markers. J. Alzheimer’s Dis..

[37] Liu J., Hjorth E., Zhu M., Calzarossa C., Samuelsson E., Schultzberg M., Åkesson E. (2013). Interplay between human microglia and neural stem/progenitor cells in an allogeneic co-culture model. J. Cell. Mol. Med..

[38] Akhter R., Shao Y., Formica S., Khrestian M., Bekris L.M. (2021). TREM2 alters the phagocytic, apoptotic and inflammatory response to Abeta(42) in HMC3 cells. Mol. Immunol..

[39] Lawrimore C.J., Coleman L.G., Zou J., Crews F.T. (2019). Ethanol Induction of Innate Immune Signals Across BV2 Microglia and SH-SY5Y Neuroblastoma Involves Induction of IL-4 and IL-13. Brain Sci..

[40] Lunghi G., Fazzari M., Di Biase E., Mauri L., Sonnino S., Chiricozzi E. (2020). Modulation of calcium signaling depends on the oligosaccharide of GM1 in Neuro2a mouse neuroblastoma cells. Glycoconj. J..

[41] Chester M.A. (1998). IUPAC-IUB Joint Commission on Biochemical Nomenclature (JCBN). Nomenclature of glycolipids--recommendations 1997. Eur. J. Biochem..

